# Spatially oriented plasmonic ‘nanograter’ structures

**DOI:** 10.1038/srep28764

**Published:** 2016-06-30

**Authors:** Zhe Liu, Ajuan Cui, Zhijie Gong, Hongqiang Li, Xiaoxiang Xia, Tiehan H. Shen, Junjie Li, Haifang Yang, Wuxia Li, Changzhi Gu

**Affiliations:** 1 Beijing National Laboratory for Condensed Matter Physics, Institute of Physics, Chinese Academy of Sciences, Beijing 100190, China; 2 College of Materials Science and Engineering, Beijing University of Technology, Beijing 100124, China; 3 School of Physics Science and Engineering, Tongji University, Shanghai, 200092, China; 4 Joule Physics Laboratory, School of Computing, Science and Engineering, University of Salford, Salford, M5 4WT, UK; 5 Collaborative Innovation Center of Quantum Matter, Beijing 100871, China

## Abstract

One of the key motivations in producing 3D structures has always been the realization of metamaterials with effective constituent properties that can be tuned in all propagation directions at various frequencies. Here, we report the investigation of spatially oriented “Nanograter” structures with orientation-dependent responses over a wide spectrum by focused-ion-beam based patterning and folding of thin film nanostructures. Au nano units of different shapes, standing along specifically designated orientations, were fabricated. Experimental measurements and simulation results show that such structures offer an additional degree of freedom for adjusting optical properties with the angle of inclination, in additional to the size of the structures. The response frequency can be varied in a wide range (8 μm–14 μm) by the spatial orientation (0°–180°) of the structures, transforming the response from magnetic into electric coupling. This may open up prospects for the fabrication of 3D nanostructures as optical interconnects, focusing elements and logic elements, moving toward the realization of 3D optical circuits.

Metamaterials (MMs) have attracted enormous research interest in the past decades^1^,^2^,^3^. The electromagnetic responses of MMs are highly related to the geometric configuration of constitutional units as well as the dielectric environment, which could be modified and utilized to tailor the macroscopic properties of metamaterials. In particular, one of the key motivations in producing MM structures has always been the realization of 3D metamaterials with effective constituent properties that can be tuned in all propagation directions at various frequencies^4^,^5^. Tunable metamaterials usually refer to artificial structures that persist continuously changeable optical properties when encountered with an external influence or signal^6^,^7^,^8^,^9^,^10^,^11^,^12^,^13^,^14^,^15^,^16^,^17^,^18^,^19^,^20^,^21^,^22^,^23^,^24^, which opens exciting opportunities for construction and application in multifunctional optical devices.

The responses of metamaterials can be influenced by a variety of factors, which may come from the structure itself, the environment or the excitation source. Fundamentally, the configuration of metamaterial, either shape^6^, size^7^, arrangement^8^ or spatial orientation^9^,^10^,^11^, can be used to alter the responses directly. Meanwhile, dielectric environment, such as semiconductor substrate with carrier dissipation^12^,^13^,^14^, liquid crystal molecules^15^,^16^ or VO_2_ phase-change material^17^,^18^, have been used for electromagnetic response modulation by external signals including applied voltage, temperature variation and laser irradiation. In addition, the responses of metamaterials can be modulated by the input power of the incident electromagnetic waves such as modification of the magnetic responses of split ring resonators by implanting varactor on the ring gap^19^,^20^, or by other external excitation signals^21^,^22^,^23^,^24^.

However, fabrication of freely designable structural configuration requires dedicated fabrication process, and the controllable wavelength range is usually small due to the limited configuration variation. Recently, we have developed a method which uses focused-ion-beam irradiation for post-growth shape manipulation of metallic nanowire and thin film structures^25^,^26^,^27^. The orientation of such structures can possibly be controlled by heat^28^ or other signals, indicating multiple adjusting capability. Meanwhile, due to the nanoscale resolution and spatial precision of focused-ion-beam techniques, this method can potentially be used for producing 3D structures operating in a range of frequency bands, ranging from terahertz with feature size of tens of microns to optical regimes of hundreds of nanometers. Using this method, 3D metallic structures with readily designed orientation, are expected to provide an additional degree of freedom for adjusting the responses in a wide spectrum range.

Following our recent work on the exploration of unusual Fano resonances of ‘nanograter’ structures^27^, we further report the achievement of orientation-dependent responses over a wide spectrum with such spatially oriented 3D structures. Experimental measurements and simulation results show that such structures offer an additional degree of freedom for adjusting optical properties with the angle of the inclination of the structures, rather than their size. Typical “nanograter” structure shows non-complementary properties in transmission and reflection spectra due to the asymmetry in the direction that perpendicular to the in-plane film. Traditional magnetic responses with adjustable intensity were achieved when the H-field of the incident light passed through the SRRs. The peak response varied from 8 µm to 14 µm with the change of the spatial orientation of the structures when the H-field of the incident light was applied parallel to the SRRs. Surface current analysis showed that the large shift in frequency arose from the coupling between the freely oriented SRRs and the planar CSRR (complementary SRR), and the response type changed from a magnetic resonance to an electric resonance when altering the angle of inclination, indicating a more versatile approach to obtain tunable optical properties. This demonstrates that the structural orientation could be a potential parameter to be utilized for manipulating light propagation and opens up prospects for the fabrication of 3D nanostructures as optical interconnects, focusing elements and logic elements.

## Results

### Construction of spatially oriented 3D metallic structures

**Figure 1:**
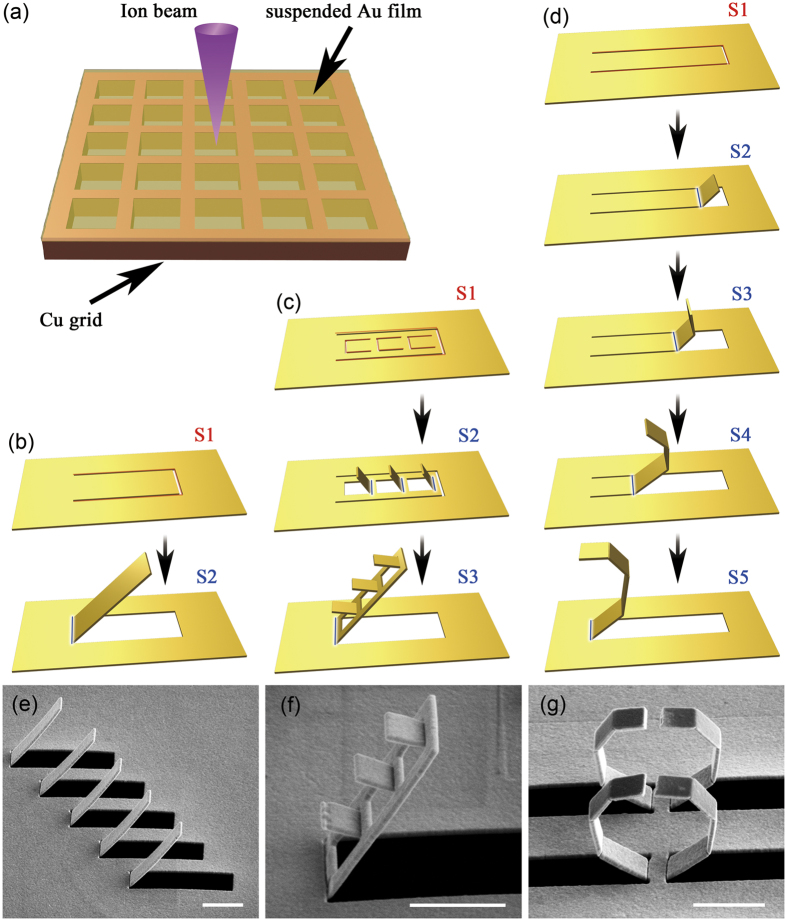
Fabrication of spatially orientated 3D structures by focused-ion-beam folding: (**a**) A suspend Au film on Cu grid. (**b–d**) Single-folding, double-folding and multi-folding process. (**e–g**) SEM images of structures achieved with single, double and multi-folding strategy, respectively. The scale bar is 2 μm.

A schematic of the fabrication strategies for 3D plasmonic structures, e.g. single-folding, double-folding and multi-folding is illustrated in Fig. 1. First, the metal film is released from the substrate layer and then transferred onto a copper grid. Self-supported metal film is obtained as shown in Fig. 1a. The effective total size of the pattern area is determined by the size of the hollow grid, which can be chosen flexibly. Then focused-ion-beam is applied onto the film to: (i) cut through the metal film for in-plane cantilever-structures with only one edge remaining connected to the rest of the film as shown in Fig. 1b–d (red path labelled S1); (ii) scan along specific path to fold the cantilever to a certain inclined angle (blue path). For single-folding (Fig. 1b), a line scanning along the cantilever base is processed to simply fold it up (labelled S2). For a typical double-folding (Fig. 1c), firstly, smaller size cantilevers are cut on a larger size cantilever, then line scans are performed on each small cantilever to fold them up, followed by an extra line scanning across the base of the large cantilever, by which a hierarchy folding is achieved as shown in Fig. 1c(S2–S3). Figure 1d shows one of the multi-folding designs, which is carried out by multiple steps (S2–S5) scanning along one direction of the cantilever. The corresponding scanning electron microscope (SEM) images of structures obtained by the aforementioned three kinds of strategies are presented in Fig. 1e,f,g, respectively. The specified inclined angles can be well controlled by the current, the irradiation time and the incident direction of the ion beam^25^,^26^.

**Figure 2:**
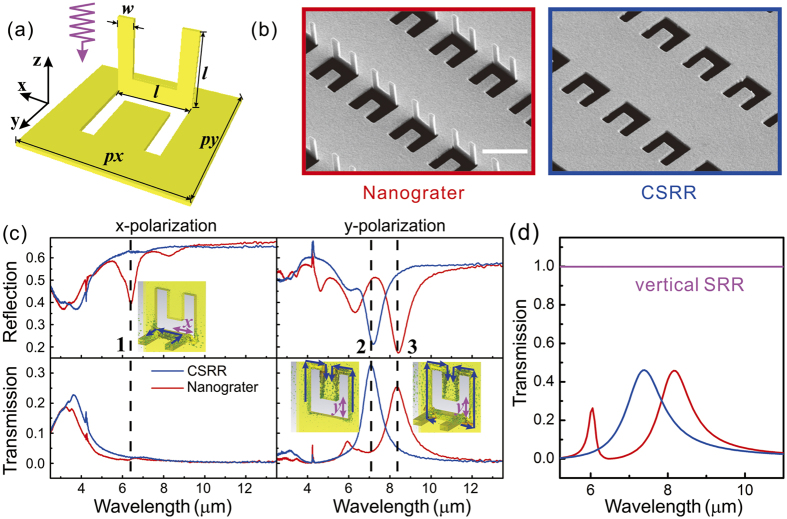
The reflection and transmission spectra of planar CSRRs and nanograter composite structures with light impinging normally from positive *z* direction: (**a**) Schematic of nanograter structures. (**b**) SEM images of nanograter and CSRR structures, the scale bar is 2 μm. (**c**) Reflection and transmission spectra obtained by FT-IR spectrometer with *x* and *y*-polarized incidences, the insets are the simulated current distributions at the wavelength of the resonance peaks. The direction and length of the arrows indicate the direction and relative value of the surface current density. (**d**) Simulated transmission spectra of the nanograter (red), planar CSRR (blue) and vertical SRR arrays (pink) with *y*-polarized incidence.

It can be well inferred from the fabrication process that when a cantilever is folded, a complementary hole-shape is left in the original metal film to form directly the composite structures. As illustrated in Fig. 2a, the 3D structure contains a vertical SRR and a planar CSRR. SRR is a typical element for magnetic response where surface current loop can be coupled^29^,^30^, while in CSRR the waveguide mode can be excited^31^. In that, we expect the combination of these two structures to be a useful way to investigate their coupling effect.

### Optical characteristics of ‘nanograter’ and planar CSRR structures

Arrays of 3D structures with unit size of *w* = 300 nm, *l* = 1.4 μm, *px* = 2.5 μm and *py* = 5 μm, and total area of 60 μm × 60 μm, as shown in Fig. 2b, were fabricated. The size of the SRR was a little smaller than that of the CSRR due to the loss of materials when cutting by ion beam with a line scan width of about 50 nm. In order to study the contribution of the spatially oriented SRR, a planar CSRR sample was fabricated for comparison. The transmission and reflection properties were measured by a Fourier Transform Infrared (FT-IR) spectrometer with light incidence from the vertical SRR side, and the corresponding spectra by different incidence polarization are shown in Fig. 2c. When the incident light is *x*-polarized, a response at 6.4 μm (the dashed line 1) appears in the reflection spectra of the nanograter structures (red curve) but no corresponding response in the transmission spectra. Meanwhile, neither reflection nor transmission responses is observed for planar CSRRs (blue curve). In order to understand such a behavior, the current distribution at the peak wavelength was simulated, as shown in the inset by the dashed line 1. A current loop was found on the U-shaped vertical SRR, which was excited by the magnetic field passing through the SRR directly^4^. The reflected signal comes from the current loop that radiates electromagnetic waves backward; however, the forward radiation was screened by the metal film and no transmission response can be found. For planar CSRR structures, there are neither reflection nor transmission response at this wavelength because no current loop can be excited. In short, the asymmetry of the nanograter structure showed non-complementary properties between transmission and reflection spectra, and no coupling effects were found between the vertical SRR and the planar CSRR under *x*-polarization incidence.

For *y*-polarized incidence, the spectra are quite different. Responses from both reflection and transmission have been observed, which shows complementary profiles. The response of nanograter structures (red curve at the dashed line 3) has a larger peak wavelength than that of the planar CSRRs (blue curve at the dashed line 2). The surface current loops were also simulated and shown beside the peaks. For planar CSRRs, surface currents are excited at the edge of the pattern by the electric field in *y* direction, But for nanograter structure, the surface currents distribute at the edge of the CSRR and also extend to the bottom of the vertical SRR, whose surface current has larger effective perimeter, and as a consequence, the resonance wavelength is larger^32^.

In order to further verify the effect of vertical SRR on planar CSRR, Fig. 2d shows the simulated transmission spectra of the nanograter, planar CSRR, as well as vertical SRR (although it cannot be achieved in experiment) of *y*-polarized incidence. The red and blue curves are fitted well compared to that in Fig. 2c, and for vertical SRR structure (the pink curve) the transmitted signal is almost 1.0, which means there is no resonance in this situation. In this way, it is the coupling between SRR and CSRR that dominants the peak shift compared to planar CSRR, rather than the individual property of vertical SRR. Obviously, the nearby vertical SRR provides more degrees of freedom to achieve large tunability in optical responses.

### Orientation-dependent characteristics of nanograter structures

**Figure 3:**
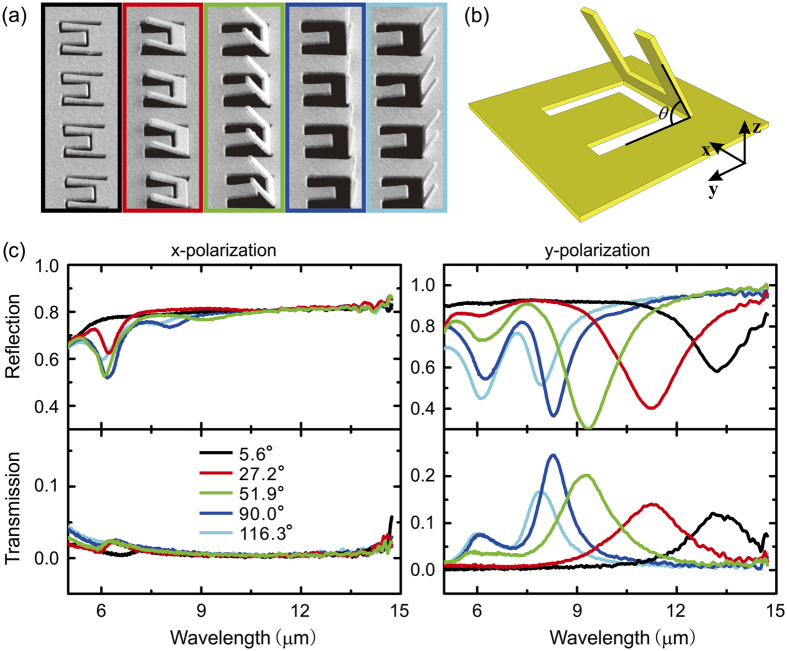
The angle of inclination dependent reflection and transmission response of nanograter structures. (**a**) SEM images of different nanograter structures; (**b**) Schematic geometry of a nanograter unit. (**c**) Reflection and transmission spectra of nanograter structures shown in (**a**).

The angle of inclination of an SRR can be precisely controlled by various fabrication parameters, e.g. the ion beam current, the effective dose, the scanning strategy and the incident direction, which provides great flexibility in controlling the orientation of the nanograter structures. Figure 3a shows a series of nanograter structures with identical sizes but different inclined angles *θ* of 5.6°, 27.2°, 51.9°, 90.0° and 116.3° (corresponding to each color frame from left to right). SEM images of patterns produced in a larger area can be found in the Supplementary Information. *θ* is defined to be the value between the planes that the SRR and CSRR located (Fig. 3b). The corresponding reflection and transmission spectra obtained by incident lights of different polarizations can be found in Fig. 3c.

**Figure 4:**
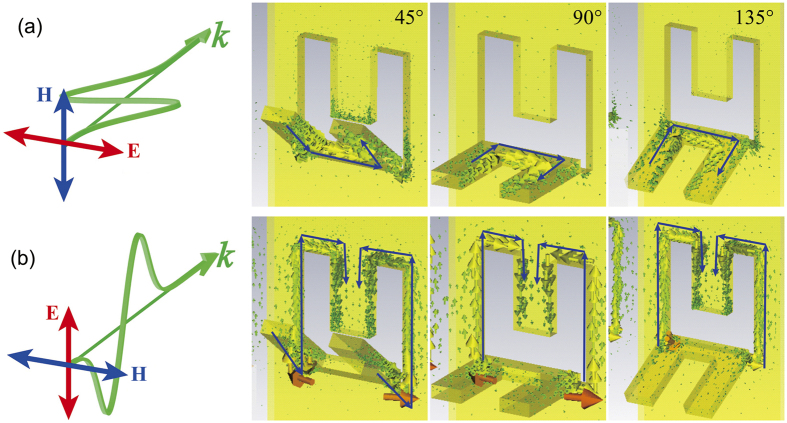
Simulated current distribution of nanograter structures: (**a**) Current distribution under *x*-polarized incidence. (**b**) Current distribution under *y*-polarized incidence.

For *x*-polarization it again shows that there are responses in the reflection spectra but not transmission. Meanwhile, the responses change very little in wavelength for different inclined angles, but have significant changes in the strength. The simulated surface currents with different inclined angles of 45°, 90° and 135° can be found in Fig. 4a. For *x*-polarization, there are surface current loops of all the three structures, which indicate that the response peak positions should be independent of the inclined angle of the SRR, but the peak amplitudes are quite different. When the angle is 90°, the magnetic field passes through the SRR directly, and the optical response has the largest amplitude of intensity (blue curve in Fig. 4c). When the inclined angle is *θ* (*θ* ≠ 90°), the magnitude of magnetic field that passes through SRR is *H* = *H*_0 _sin *θ*, where *H*_0_ is the magnetic field magnitude of the incident light. So the reflection peak intensity has maximum value at *θ* = 90°.

In the case of the *y*-polarized incidence as shown in Fig. 3c, the response peaks shift significantly from 8 μm to 14 μm with the inclined angles, which indicates the orientation-dependent controlling of the optical responses when inclined SRRs are integrated with the planar CSRRs. As can be seen from Fig. 4b, the surface currents are excited at the edge of both the SRR and CSRR, and the perimeters of the surface current decrease with inclined angles, which can qualitatively explain the peak shift in Fig. 3c. A quantitative explanation will be discussed in the following part.

### A theoretical analysis of the orientation-dependent responses

**Figure 5:**
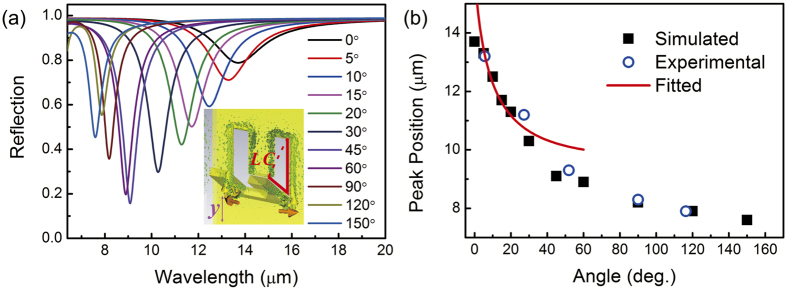
Orientation-dependent optical response of nanograter: (**a**) Reflection spectra of nanograters with different inclined angles by *y*-polarized incident. (**b**) Inclined angle dependent peak position. The black squares represent peak positions in Fig. 5a, the blue circles represent peak positions in Fig. 3c, and the red curve is the fitting based on data in Fig. 5a.

From Fig. 4b it can be seen that the current loop migrates along both the SRR and CSRR elements. When the angle of inclination reaches 90° or over, a current loop appears only around the CSRR. As the angle decreases below 90°, the current extends to the SRR, which in turn increases the effective path of the current loop. In this case, the arm of the SRR and the edge of the CSRR form a virtual split ring (inset in Fig. 5a), through which the magnetic field effectively induces a current loop that hybridizing the resonance of the SRRs and CSRRs. In principle, the current loop mimics an LC oscillator with an eigenfrequency of 33:
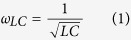
Here the capacitance of the ring, which is an unparalleled plate capacitor, can be depicted as
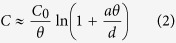
Here *θ* and *d* are the angle and distance between the two plates that formed the unparalleled plate capacitor, respectively, *a* is the side length of each plate, and *C*_0_ is the capacitance of a parallel plate condenser with the same *a* and *d*. This equation is tenable when the angle *θ* is close to 0°. In fact, the two metal plates are not actually isolated, so *d* should be treated as an effective distance. In such a system, the charges are considered to be accumulated on the portion of the metal plates that are separated by a distance greater than *d*, whereas the portions of the metal that are closer should be treated as conductive wires.

As to the calculation of inductance *L*, an approximate expression is given when the inclined angle *θ* approaches zero for a coil of isosceles triangle loop^34^:
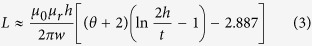
Here *w* is the width of the coil, *h* is the average length of the virtual ring arms, and *t* is the thickness of the metal film. For structures shown in Fig. 4, *h* = 1.45 μm, *t* = 0.08 μm, then according to Eqs (1, 2, 3), the response wavelength of the virtual split ring can be expressed as
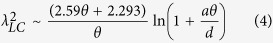


A numerical simulation of the inclined angle dependent reflection spectra of the nanograter structures was performed, as shown in Fig. 5a. A blue shift can be observed as expected when the angle increases. Figure 5b shows the values of the peak positions obtained from calculation (black square) and experiment (blue circle), and the red line is the fitted data according to Eq. (4) (the value of *a/d* derived from the fitting is 57.7). For valid approximation, data with small angles ranging from 0° to 20° were used for the fitting. It can be seen that there is an abrupt blue shift when the angle increases from 0° to about 20°; when the angle exceeds 20°, the peak position keeps moving towards the shorter wavelengths, but in a much milder way. As the angle approaches 180°, the performance of such nanograter structures becomes similar to that of planar CSRR structures.

This orientation-dependent process of the peak-shift can be considered as a transition between two modes. For inclination smaller than 90°, the magnetic response is excited by the magnetic field of the incident light, in which case the resonance can be well described by an LC model. When the angle exceeds 90°, the effect of SRR on the composite structure is weakened, and the current around CSRR is excited mainly by the electric field in the *y*-direction, so this is an electrically coupled response mode. When the inclination approaches 90°, a transition between the two modes occurs. In short, the response is closely related to the current loop excited by either the magnetic field (for angles below 90°) or the electric field (for angles above 90°), and the corresponding resonance peaks can be adjusted in a broad region ranging from 8 μm to 14 μm.

It should be noted from Fig. 4b that the surface current distributes not only on the outer edge of CSRR, but also on the inner edges. This changes the effective perimeter of surface current, thus the shape of CSRR exhibits remarkable effect on the corresponding optical resonance. Further discussion on the shape of SRR and CSRR can be found from the Supplementary Information, which shows that it is the shape of CSRR rather than inclined SRR that mainly affects the resonance frequency of the composite structure. When rectangular holes were used instead of CSRRs, the value of *h* and *a/d* in the LC model discussed above should be changed accordingly.

## Discussion

The results presented suggest that the spatially oriented nanograter structures can potentially be used as optical devices. The frequency of response can be flexibly modified by the orientation of SRRs in those nanograter structures. A wide range of frequency shift in infrared region from 8 μm to 14 μm was also obtained using a theoretical model and in good agreement with the experimental observations. The orientation-dependent response can be interpreted by the mode transition from magnetic dipole to electric dipole. Whilst the orientation of SRR can be determined by the focused-ion-beam, it may also be possible by other means such as heat or pressure. It is expected that such structures may offer the potential to explore a large variety of novel optical applications associated with the tuning of the frequency of responses, as well as an additional approach for further cutting-edge downscaling of 3D nano-metamaterials for applications of passive and active optical devices, directional antennas, light focusing and light isolation devices, owing to its asymmetry characteristics with respect to the direction of the propagation of the light beam.

## Methods

### Sample Preparation

Photoresist (S1813) was spin coated on silicon wafer and baked at 115 °C for 2 min, followed by magnetron sputtering deposition of Au film. The samples were immersed into acetone for 24 h to fully dissolve the resist, and a copper grid (Beijing Zhongjingkeyi Technology Co., Ltd) with a hole-size of 100 μm was used to pick up the Au film that suspended in acetone and then dried in N_2_ to get a flat and self-supported Au film. An FIB system (FEI Helios 600i) was used to, first cut through the metal film making patterns that connects to the metal film, and then scan the bottom of the patterns to bend it up. The acceleration voltage of Ga^+^ is 30 keV, and the ion beam current is 30 pA. The area of the patterns is about 60 μm × 60 μm.

### Optical measurements

Transmission/reflection signals were collected by an optical microscope (Hyperion2000), and the spectra were measured by Fourier-transform infrared spectrometer (Vertex 70, Bruker) using a ×36, 0.5 numerical aperture objective, and a home-made pinhole setup to confine the incident angle of light^35^. Reflection spectra were calibrated using measurements of a silver mirror, while the transmission spectra were calibrated using air as a reference.

### Numerical simulation

The reflection spectra and surface current distributions were simulated using a commercial software package CST Microwave Studio. Realistic parameters were used to describe gold’s properties: electric conductivity of 4.561 × 10^7^ S/m. The reflection spectra were simulated by a frequency domain solver with unit cell boundary conditions, and the surface current distributions were obtained by H-field/surface current monitor.

## Additional Information

**How to cite this article**: Liu, Z. *et al*. Spatially oriented plasmonic ‘nanograter’ structures. *Sci. Rep.* **6**, 28764; doi: 10.1038/srep28764 (2016).

## Supplementary Material

Supplementary Information
