# Supplementary material for: Spatially oriented plasmonic ‘nanograter’ structures
Source: Sci Rep. 2016 Jun 30;6:28764. doi: 10.1038/srep28764 (PMC4928092; doi:10.1038/srep28764)
Supplement: Supplementary Information [file srep28764-s1.doc]

**Spatially oriented plasmonic ‘nanograter’ structures**

Zhe Liu^1†^, Ajuan Cui[[1]](#footnote-2),2†, Zhijie Gong^3^, Hongqiang Li^3^, Xiaoxiang Xia^1^, Tiehan H. Shen^4^,Junjie Li^1^, Haifang Yang^1^, Wuxia Li^1*^ and Changzhi Gu^1,5*^

^1^Beijing National Laboratory for Condensed Matter Physics, Institute of Physics, Chinese Academy of Sciences, Beijing 100190, China

^2^College of Materials Science and Engineering, Beijing University of Technology, Beijing

100124, China

^3^School of Physics Science and Engineering, Tongji University, Shanghai, 200092, China

^4^Joule Physics Laboratory, School of Computing, Science and Engineering, University of Salford, Salford, M5 4WT, UK

^5^Collaborative Innovation Center of Quantum Matter, Beijing 100871, China.

^†^ These authors contributed equally to this work.

^*^Authors to whom correspondence should be addressed. E-mail: liwuxia@aphy.iphy.ac.cn; czgu@aphy.iphy.ac.cn

Nanograter arrays with various angles of inclination are shown in Figure S1 (a). Structures with array size of 60 μm × 60 μm were fabricated. Larger area views are displayed in Figure S1 (b) and (c). Typically structures in an area with size of 1 mm × 1 mm can be fabricated in about 5 hours.

**
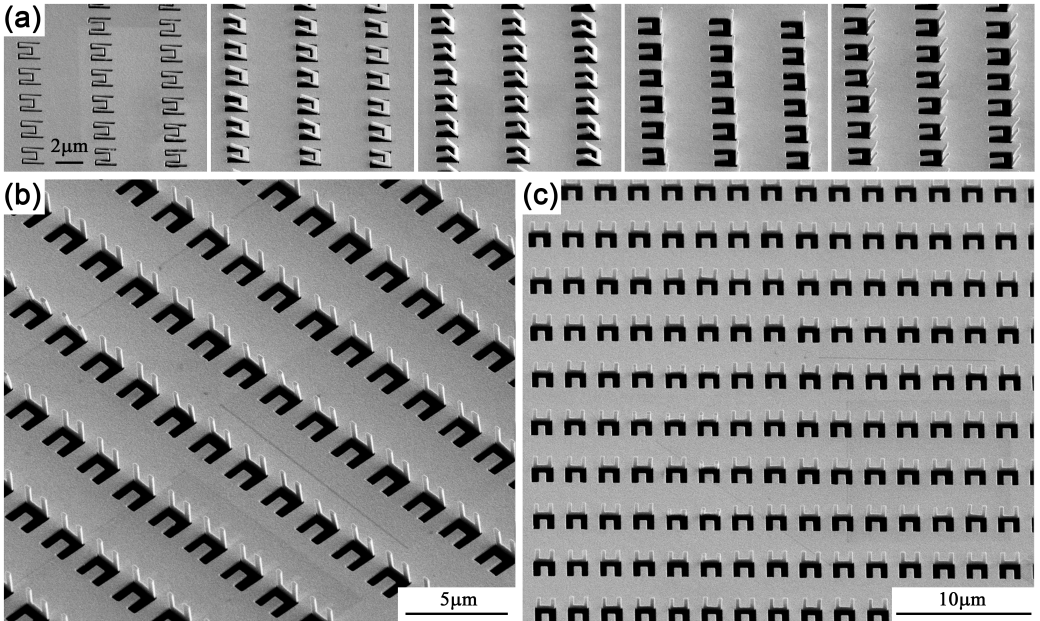
**

**Figure S1.** SEM images (viewing angle of 45°) of nanograter arrays with various angles of inclination: (a) 5.6°, 27.2°, 51.9°, 90.0° and 116.3° from left to right, respectively; (b) and (c) larger area views of vertically oriented structure arrays.

Whilst the preset angle of inclination provides a very effective way to alter the spectral response, other changes in the geometry of the nanograter naturally also affect the optical response. Fig. S2 shows the simulation results for three contrasting structures, they are SRR-CSRR, SRR-hole and square-hole, and all have an angle of inclination of 45°.

In addition, the response controllability of the inclined angle of the SRR-CSRR and square-hole structures was compared the simulated results are shown in Figure S3. It can be seen that the square-hole structure exhibits a narrower response range, which only changed from 10 μm to 6.4 μm. Such phenomena are mainly due to the difference in surface current distributions for different structures, which inspires an optimized structure design for property control.

**
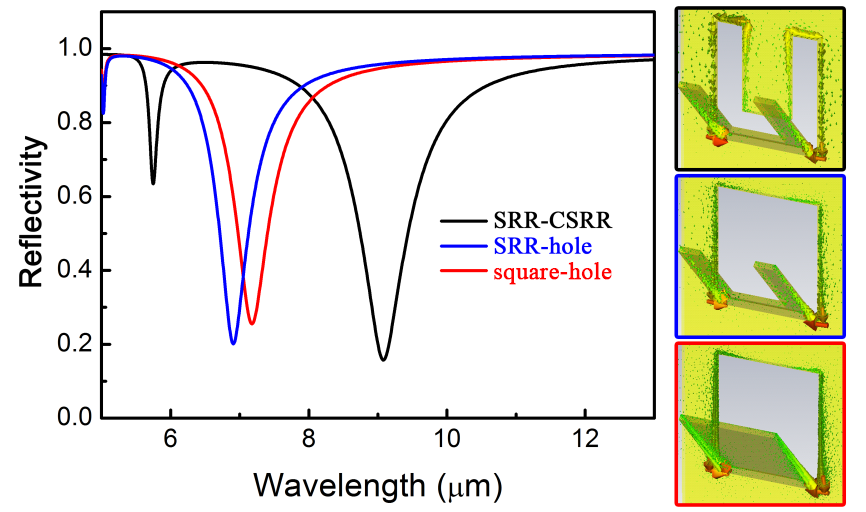
**

**Figure** **S2.** Simulated reflection spectra of structures with the angle of inclination of 45° under *y*-polarized incidence. Black curve: inclined SRR and planar CSRR; Blue curve: inclined SRR and planar hole; Red curve: inclined square unit and planar hole. The related surface current distributions are displayed on the right side.

**
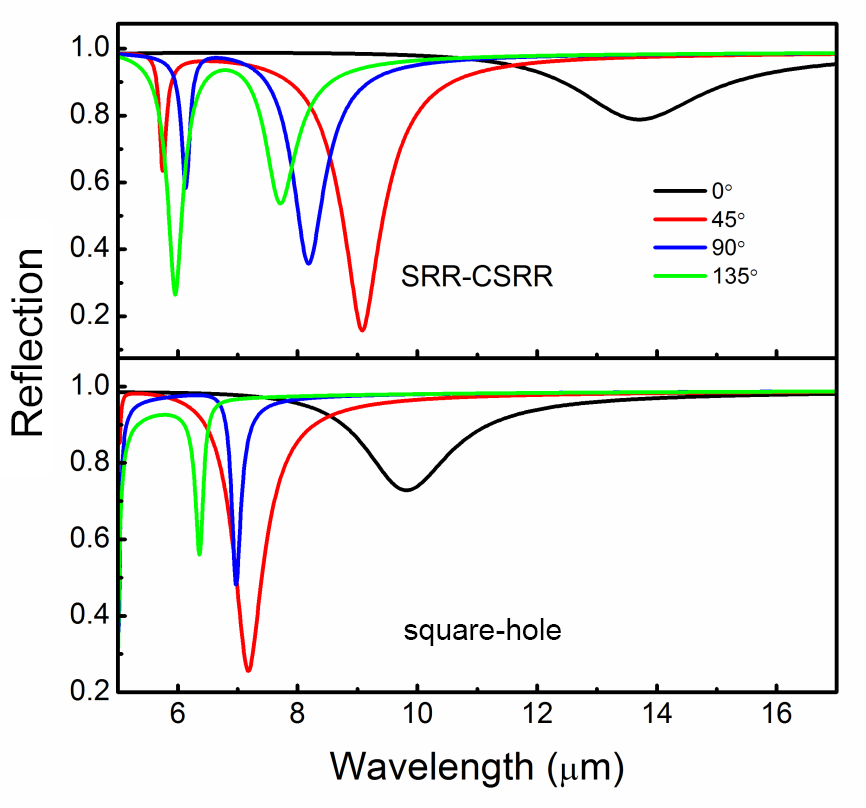
**

**Figure S3.** Simulated reflection spectra of the SRR-CSRR and square-hole structures with the angle of inclination of 0°, 45°, 90° and 135°.

1. [↑](#footnote-ref-2)
